# Odontological Society of Pennsylvania

**Published:** 1889-02

**Authors:** 


### PROCEEDINGS.

### The Odontological Society of Pennsylvania.

FROM THE NOTEBOOK OF A DENTAL STUDENT.

[Tenth Annual Meeting December 12th and 13th, 1888.]

The excellent programme announced by this flourishing society was most admirably carried out in nearly every particular. Papers, discussions, clinics, and displays were of the highest order of excellence.

*Dr. Chas. J. Essig*—In his opening address offered encouraging words to the younger members of the profession. He said that the older members often rob the younger ones of what are really their own original ideas by calling attention to something similar evolved by themselves in years gone by. He urged the importance of more systematic methods in education.

*Dr. A. L. Northrop*—In his response spoke of the objects and purposes of dental societies. He thought that when the real object is, as it should be, the advancement of the profession rather than of individuals, but one society will be found, as is the case in New York City and Philadelphia.

Dental societies and dental colleges go hand in hand. The societies are continually demanding higher education, and in response to this demand the colleges give it. The great need in society meetings is greater concentration of thought. In the discussions we wander too far from the subject under consideration, and bring in too many side issues.

In addition to this one society for scientific and practical work, social clubs and friendly gatherings bring men nearer to one another and cultivate feelings of harmony.

*Dr. Geo. S. Allan*—Read a paper on the Etiology of Dental Caries, taking the ground that, although poor morphological outline, malformation, malposition, and poor structure invite caries, they do not produce it. Acids are recognized to be the real cause of caries, lactic acid secreted by microbes being the main factor in the work of destruction.

*Dr. Jas. Truman*—Read a paper on the Treatment and Filling of Root Canals. He said that the dental tissues are so intimately connected that what affects one affects all; he has little hope of saving pulps that have once been in a pathological condition.

*Dr. Perry*—Fills the apex with gold whenever possible, and fills the canal with oxy-chloride. Cotton was advocated by several speakers, as a root-filling, especially when saturated with iodoform disguised with oil of cloves.

*Dr. Allan*—Uses cotton saturated with bichloride of mercury and dipped in resin dissolved in ether.

*Dr. Crouse*—Said of the *Essayist*, that he was surprised to find that his old teacher had not learned any thing in the last twenty-five years, and was sorry that he had so little faith in pulp capping. He expects to save seventy-five per cent. of free exposures, using carbolic acid to cook the albumen on the surface of the pulp, capping with oxy-chloride of zinc and filling at once.

*Dr. Atkinson*—Said that he saves all but two classes of exposed pulps—your correspondent did not catch clearly the first class—the second is when pulp stones exist. He said that one cause of failure is from not knowing how remedies act. A man may follow closely the directions of one who is very successful in saving pulps, but if he has not the same degree of accurate skill, he will not succeed, though he may seemingly do every thing in the way he is told.

*Dr. Dwinelle*—Would give the pulp every chance.

The evening session was devoted to a lantern exhibit by Drs. Allan, Andrews, and Sudduth, with an introductory lecture by Dr. R. R. Andrews and explanatory remarks by Dr. Sudduth. Micro-organisms *in situ* in carious dentine were shown clearly on the screen, and the same organisms were shown in artificial caries produced by the methods of Dr. Miller, of Berlin, proving the correctness of his views.

The exhibit was admitted to be the finest ever made, though exception was taken to some of their interpretations, especially by Dr. Atkinson.

*Dr. T. S. Waters*—Read a paper on Removable Crown and Bridgework.

*Dr. Dwinelle*—Opened the discussion of the paper, claiming that while Dr. Waters was the inventor of *removable* bridgework, he, Dr. D., was the first to use crowns and bridges.

*Dr. Crouse*—Said that he had never seen a perfectly satisfactory piece of removable bridgework. He would always be afraid it would get into the throat as small partial plates used to do. Also removing and replacing is liable to loosen the teeth of attachment.

*Dr. Atkinson*—Said he had seen bridges put on teeth that were literally dancing in quagmires of pus, which being held steady by the bridge, became firm, strong, and healthy. He said he did not mean to imply by this that it was not necessary to treat roots and make them healthy first, but that it showed how essential it is that they should be held immovably in place.

*Dr. Bennett*—Puts a cylinder in the root, but instead of using a split pin, he roughens the pin and inserts it with gutta-percha. It can be removed for repairs if necessary, but is not removable by the patient, and is in no danger of being swallowed. He prefers to have the porcelain sit squarely against the gum tissue.

*Dr. S. E. Perry*—Read a paper on the Treatment of Approximal Surfaces. He said that in his younger days he had been, as he now is, a contour operator but was led astray by Dr. Arthur. He found that under the Arthur system his work failed exceedingly often, while he had had reasonably few failures by contouring; he returned to his first love. He found that the shoulder left at the gum to hold the teeth apart would decay and the teeth come together again with flat surfaces of unprotected dentine. It is much easier to cut away the side of a tooth and leave only a small cavity to be filled, which would otherwise require a large contour filling, but it will not save the tooth. He does not believe in building out knobs of gold to hold the teeth apart, but he does believe in restoring the teeth to their natural form, which is different in every mouth. When he has small approximal cavities to fill he uses small pieces of crystal gold, and small bell-shaped instruments. He said—only the other day a lady came to me and said "Doctor, do you remember, many years ago, when you were filling some of my teeth you wanted to file between these molars, because you thought there either was or would be decay, and mother begged you not to do it? Well! nothing has ever been done to them, in all these years, and they are as good as ever. Mother knew, didn't she?" And I tell you mothers do know, and the public in general knows. They call it *taking the enamel off the teeth*; and it is something more than that; it is taking away from a man that peaceful conscience which he enjoys when he contours properly, and instead of trying to improve on the work of the Creator simply copies nature.

*Dr. Dwinelle*—Said that he was the originator of contour fillings, and had advocated it among dentists who criticized him severely. He remembers when the manufacturers were trying hard to keep their gold from being *sticky*. Dr. Dwinelle thought is would be better if students were not allowed to use the dental engine during their first session, as hand excavators developed finer delicacy of touch.

*Dr. S. H. Guilford*—Read a paper on Voluntary Tooth Movement Causing Abnormal Interdental Spaces. He thought this was due to calculus or the roots; Dr. Atkinson thinks it is caused by Rigg's disease; Drs. Sudduth and Truman do not think it due to Rigg's disease as no pus was visible to the naked eye, though neither the microscope nor peroxide of hydrogen was used to detect it.

Thursday afternoon Dr. McLain gave an exhibition of his method of sharpening instruments, using a metal disk to strengthen an emery disk of large size run in the dental engine. To sharpen a round edge he places a rubber disk between the emery and metal, forming a cushion which works like a charm.

*Dr. J. A. Woodward*—Exhibited his Mouth Illuminating Apparatus; Dr. Morey demonstrated his method of making a cap for a root all of one piece of metal and without the use of solder. Dr. Morey also demonstrated the use of his compound-lever, self-adjusting, separators. The writer, having had his own right superior central and lateral incisors—which would not admit a sheet of paper—easily separated to the space of eight sheets, pronounces it *a daisy* for both patient and operator.

*Dr. E. T. Starr*—Inserted two of Dr. How's Porcelain Inlays, which were much admired by all who saw the completed operation. Dr. T. S. Waters and Dr. H. A. Parr exhibited removable bridgework.

*Dr. H. C. Register*—Made a large contour filling in an upper central incisor using his dental engine, plugger and mallet.

Attractive exhibits were made by Justi, White, Williams, Welch, Gideon Sibley, the Wilmington Manf. Co., and Parke Davis & Co

*Dr. J. N. Crouse*—Presented to the association the Constitution and By-Laws of the Dental Protective Association, of which he is Chairman. The object of the association commends itself to all dentists, being their protection against exactions and prosecutions—more especially at this time the suits of the International Tooth Crown Co., against those engaged in crown and bridgework. Liberal contributions should be made to the general fund for this purpose, of which Lyman J. Gage, Vice-President First National Bank, Chicago, is Treasurer.

